# Murine but Not Human Basophil Undergoes Cell-Specific Proteolysis of a Major Endoplasmic Reticulum Chaperone

**DOI:** 10.1371/journal.pone.0039442

**Published:** 2012-06-18

**Authors:** Bei Liu, Matthew Staron, Zihai Li

**Affiliations:** Department of Microbiology and Immunology, Hollings Cancer Center, Medical University of South Carolina (MUSC), Charleston, South Carolina, United States of America; Instituto de Biofisica Carlos Chagas Filho, Universidade Federal do Rio de Janeiro, Brazil

## Abstract

**Introduction:**

Basophil has been implicated in anti-parasite defense, allergy and in polarizing T_H_2 response. Mouse model has been commonly used to study basophil function although the difference between human and mouse basophils is underappreciated. As an essential chaperone for multiple Toll-like receptors and integrins in the endoplasmic reticulum, gp96 also participates in general protein homeostasis and in the ER unfolded protein response to ensure cell survival during stress. The roles of gp96 in basophil development are unknown.

**Methods:**

We genetically delete gp96 in mice and examined the expression of gp96 in basophils by Western blot and flow cytometry. We compared the expression pattern of gp96 between human and mouse basophils.

**Results:**

We found that gp96 was dispensable for murine basophil development. Moreover, gp96 was cleaved by serine protease(s) in murine but not human basophils leading to accumulation of a nun-functional N-terminal ∼50 kDa fragment and striking induction of the unfolded protein response. The alteration of gp96 was unique to basophils and was not observed in any other cell types including mast cells. We also demonstrated that the ectopic expression of a mouse-specific tryptase mMCP11 does not lead to gp96 cleavage in human basophils.

**Conclusions:**

Our study revealed a remarkable biochemical event of gp96 silencing in murine but not human basophils, highlighting the need for caution in using mouse models to infer the function of basophils in human immune response. Our study also reveals a novel mechanism of shutting down gp96 post-translationally in regulating its function.

## Introduction

Although contributing to less than 1% of white blood cells in the peripheral blood [1], basophils are increasingly recognized in regulating both innate and adaptive immunity [2]. They constitutively express FcεRI, CD40L and CCR3 on the cell surface. Basophils can be rapidly recruited to the lung after an allergen challenge [3]. They contribute to allergic disease by releasing inflammatory mediators (e.g., histamine, LTC4) and T_H_2-polarizing cytokine IL-4 and IL-13. They have also been implicated in directing IgE class switch independent of T cells [4]. Moreover, basophils can be directly activated by protease allergens to produce IL-4 and thymic stromal lymphopoietin, which are important for T_H_2 induction *in vivo* [5]. However, basophil study has not been free of controversies. Using genetic deletion of dendritic cells in mice, it was later found dendritic cells rather than basophils are the major antigen-presenting cells for eliciting T_H_2 responses against allergens [6] and parasites [7], [8]. Importantly, despite the increasing interests in basophil biology, the rarity of basophils in mice has long been recognized, which has raised concerns in the field on the suitability of mouse as a model to probe the physiological function of this population [9]. Provocative suggestions have been made to suggest that mouse differ from other species by having a unique cell type that “shares some characteristics of basophils (e.g,. the cell-surface marker profile) but is a nongranulated polymorphonuclear leukocyte subtype distinct from the classical metachromatically staining granulated basophil” [9].

gp96 is a major molecular chaperone in the lumen of the endoplasmic reticulum (ER). In addition to its roles in unfolded protein response (UPR) and in the general ER protein quality control, gp96 serves as a master chaperone for TLRs and integrins [10], [11], [12], [13], [14], [15]. gp96 null cells do not have functional TLRs including TLR1, TLR2, TLR4, TLR5, TLR6, TLR7 and TLR9, or integrins such as α4 and β2 integrins. Consistent with its general roles in ER function, gp96 is constitutively expressed in virtually all cell types and is transcriptionally co-regulated with GRP78 [16]. Using conditional knockout mice, we found recently that gp96 plays critical roles in early T and B lymphopoiesis [15], as well as in platelet development [17]. There are no natural mammalian mutant cells with gp96 deletion. In this study, we found, surprisingly and unexpectedly, that gp96 is dispensable for murine basophil development. The primary murine but not human basophils were devoid of intact gp96 due to proteolysis, raising an intriguing question of shutting down gp96 and its client network posttranslationally in maintaining basophil function. The striking difference between human and murine basophils highlights the need for caution to use murine models to infer the function of human basophils.

## Results

### Bone marrow antigen capturing cells are basophils

**Figure 1:**
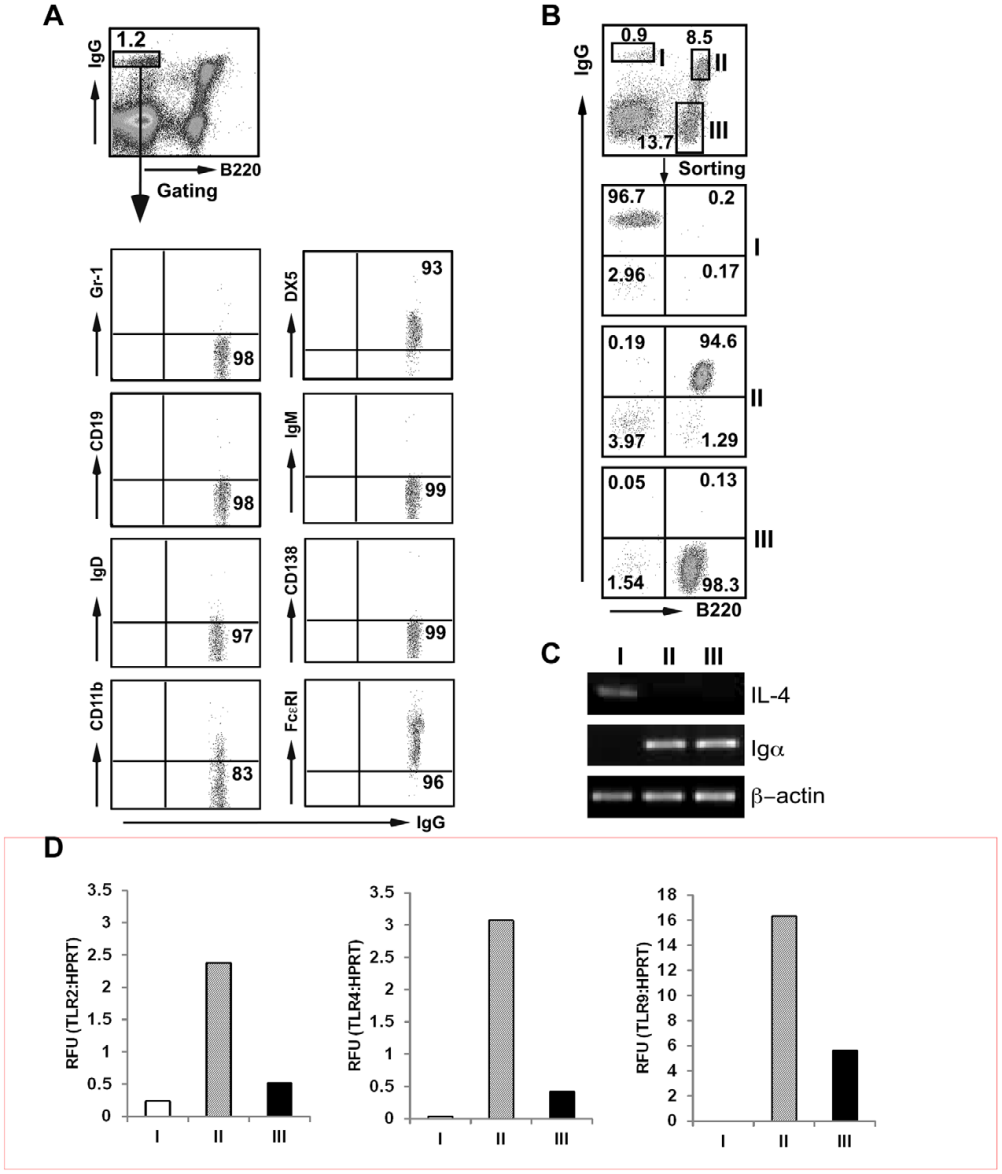
Bone marrow B220^−^IgG^high^ cells are basophils. *A*, Phenotype of B220^−^IgG^high^ cells. Numbers represent percentages of gated population or corresponding quadrants. *B*, Phenotypic analysis of FACS sorted BM cells based on cell surface markers of B220 and IgG. (C) RT-PCR analysis of IL-4 and Igα mRNA.

In 2000, McHeyzer-Williams *et* *al.* described a population of B220^−^CD19^−^Ag^+^ “memory B cells” in the bone marrow [18]. However, Bell and Gray found that this cell type did not represent B cell but an antigen (Ag)-capturing cell due to the abundant expression of high affinity Fc γ receptor CD64 (FcγRI) [19]. Mack *et* *al.* later argued that this Ag-capturing cell is actually a basophil based on surface expression of IgE, IgG, IL-3R, CD16/32, high affinity Fc ε receptor (FcεRI) and its ability to produce large amount of IL-4 upon cross-linking of cell surface Igs [20]. The ability to capture Ag is dependent on surface IgE receptors. We confirmed indeed that B220^−^IgG^high^ population in both BM and spleen were basophils based on: (i) cell surface marker of B220^−^IgG^+^CD19^−^IgM^−^IgD^−^CD138^−^Gr-1^−^DX5^+^FcεRI^+^CD11b^low^ by flow cytometry (Fig. 1A); and (ii) expression of IL-4 but not B cell receptor (BCR) component Igα of sorted population (population I) by RT-PCR (Fig. 1B and Fig. 1C). Whether basophils should be more suitably defined by cell surface marker or morphology remains an unsettling matter [9] (see later for further discussion). Although it has been shown that human basophils express both TLR2 and TLR4 mRNA [21], we found that murine basophils express very low level of mRNA for TLR2, TLR4 and TLR9 by qRT-PCR (Fig. 1D).

### Basophil develops in the absence of gp96

**Figure 2:**
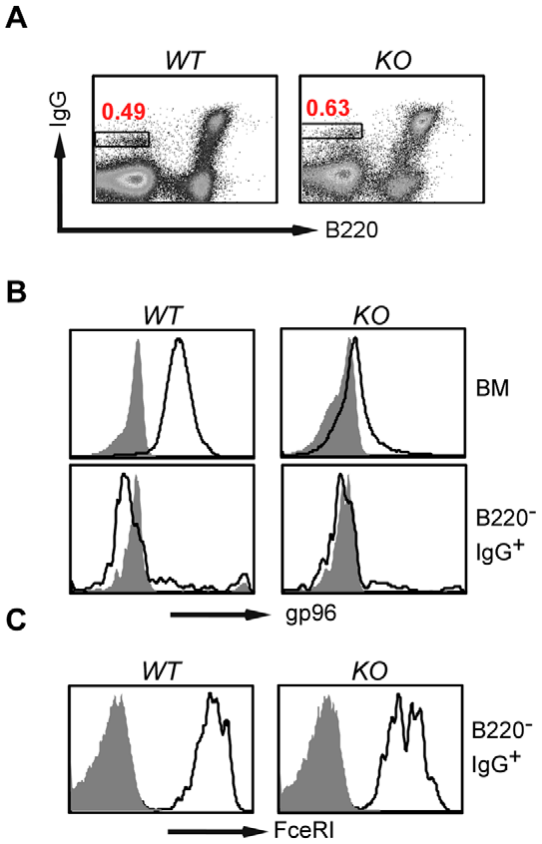
Basophil develops in the absence of gp96. *A*, Presence of B220^−^IgG^high^ basophil population (boxed) in the bone marrow of gp96 *KO* mice. *B*, Intracellular stain demonstrated an efficient deletion of gp96 in bone marrow cells of *Hsp90b1^flox^Rosa26^ERcre^* (*KO*) mice by TAM. *C*, Both *WT* and gp96 *KO* B220^−^IgG^high^ basophils express FcεRI (open histogram indicates gp96 or FcεRI; shaded histogram, isotype control).

To address the roles of gp96 in basophil development, we crossed *Hsp90b1^flox^* mice (a conditional gp96-deficient mouse by inserting a loxP sequence at each side of the first exon of gp96-encoding gene *Hsp90b1*) with *Rosa26^ERcre^* mice and induced gp96 deletion from all lineages of hematopoietic cells including basophils with Tamoxifen (TAM) [15]. By intracellular stain, we found that gp96 was efficiently deleted from bone marrow cells (BM) of gp96 knockout (*KO*) mice (Fig. 2), which was further confirmed by Western blot using gp96 antibody (data not shown). We found that BM FcεRI^+^B220^−^IgG^high^ basophil population was readily detectable in *KO* mice (Fig. 2), indicating that basophil development could proceed well in the absence of gp96. There was no evidence of basophilia in gp96 *KO* mice, suggesting that gp96 is dispensable for the development and homeostasis of basophils.

### Primary basophils do not express intact gp96

**Figure 3:**
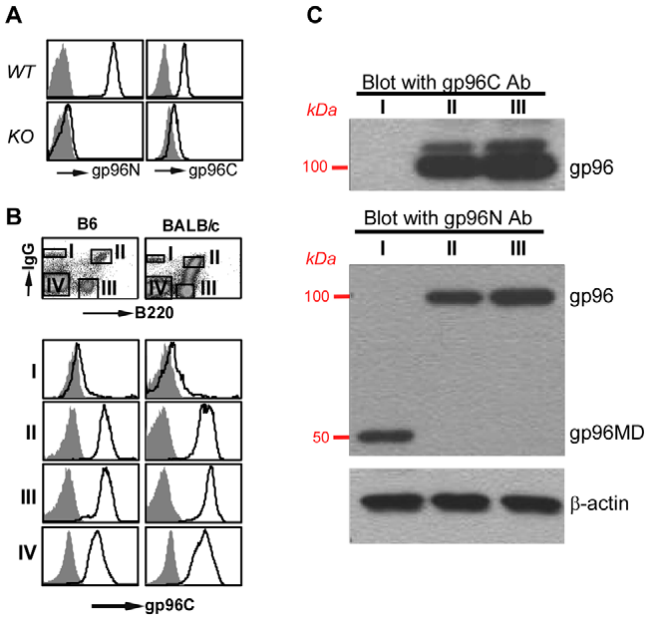
Basophil does not express full-length gp96. *A*, Intracellular stain of *WT* and *KO* B cells with isotype control (shaded histogram) or antibody against gp96N and gp96C (open histogram). *B*, Basophil (population I) does not express gp96C (open histogram). Shaded histograms are staining result with isotype control antibody. *C*, Western blot for gp96N and gp96C of sorted populations. β–actin was blotted to indicate equal loading of cell lysates.

To further probe the roles of gp96 in basophil function, we examined the level of gp96 expression in various cellular compartment of the hematopoietic system including basophils in wild type (*WT*) mice by intracellular stain using two gp96 antibodies (Abs): gp96N Ab (clone 9G10) (recognizing 280–344 sequence of gp96) and gp96C Ab (specific for C-terminal 787–802). Both Abs are specific for gp96 as they stain gp96 strongly in *WT* cells but not gp96 *KO* B cells (Fig. 3A). To our surprise, however, we found that gp96C Ab was unable to stain for B220^−^IgG^high^ basophil population in the *WT* bone marrow (population I), which was confirmed with multiple strains of mice including BALB/c and C57BL/6 mice (Fig. 3B). This data suggests that, in murine basophils, either full-length gp96 is not expressed or gp96 adopts a unique conformation to mask its C-terminal epitope. To differentiate these two possibilities, we performed Western blot analysis of the highly purified basophil populations after cell sorting, and found that basophils but not other cell populations expressed a shorter gp96 variant at 50 kDa, which reacted only to gp96N Ab (Fig. 3C). gp96C Ab was unable to detect any gp96 from basophils (only 90–100 kDa region of the blot was shown). The shortened gp96 molecule in basophil was named gp96MD (for mysterious and dwarf form of gp96). Extensive Western blot with gp96 antibody did not find gp96MD in any other primary mouse tissues and cells (data not shown). Thus, the presence of gp96MD is unique to basophils.

### C-terminal dimerization is essential for gp96 chaperone function

**Figure 4:**
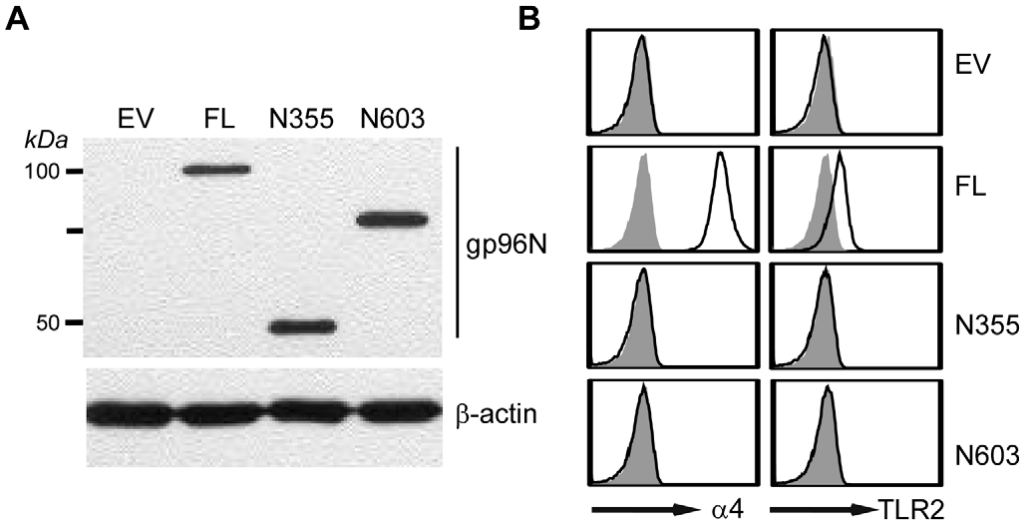
C-terminal deletion mutants of gp96 are unable to chaperone TLRs and integrins. *A*, gp96 null preB cells were transduced with retroviral expression vectors for empty vector (EV), full-length gp96 (FL) or gp96 deletion mutants (N355, N603), followed by Western blot with gp96N Ab or β–actin as a loading control. *B*, Various transfectants in (A) were analyzed by flow cytometry for cell surface expression of gp96 clientele TLR2 and α4 integrin (open histogram). Shaded histogram depicts isotype control.

Similar to its cytosolic HSP90 counterpart, gp96 contains N-terminal ATP binding/ATPase domain, followed by the middle charged domain and the C-terminal dimerization domain (DD) [16]. gp96MD is not expected to be functional due to lack of the C-terminal dimerization domain [10], although the basic chaperone unit of gp96 has been suggested to reside in the N-terminal 355 amino acids (N355) [22]. To more conclusively address the roles of gp96 C-terminal domain on its overall chaperone function, we generated retroviral expression vectors for full-length gp96 and two C-terminal deletion mutants (N355, N603). N355 and N603 denote two N-terminal gp96 fragments of 355 and 603 amino acids respectively, neither of which contain the C-terminal dimerization domain [23]. We transduced a gp96-null pre-B cell lines with empty vector (EV) or various gp96-expressing constructs. Our previous work demonstrated that gp96 client proteins do not fold properly and are unable to export to the cell surface in the absence of gp96 [11], [12]. We thus compared the cell surface expression level of TLR2 and α4 integrin on these transfectants by flow cytometry. We found that none of these mutants of gp96 were able to rescue gp96 null cells for cell surface expression of gp96 clientele (Fig. 4), demonstrating unequivocally the importance of the C-terminal domain of gp96 in its chaperone function and suggesting strongly that gp96MD is non-functional.

### Truncation of gp96 in basophil is not due to alternative splicing but is coupled with UPR

**Figure 5:**
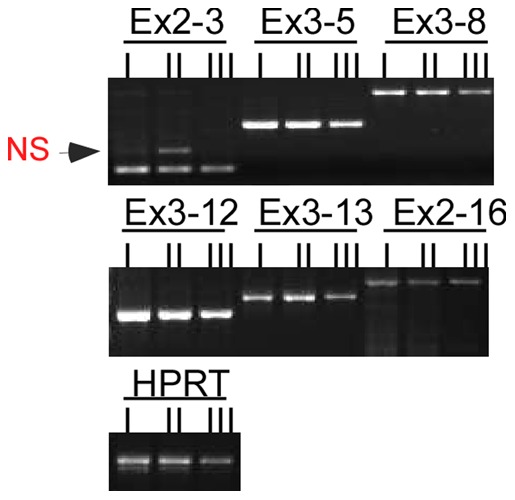
Expression of gp96MD is not due to a basophil-specific alternative splicing. Exon (Ex)-specific PCR analysis of gp96 cDNA from sorted populations of bone marrow cells. (Ex2-3, Ex3-5, Ex3-8, et al. indicates the amplified fragments between different exons; NS: non-specific band). Population I: B220^−^IgG^high^, Population II: B220^+^IgG^high^, Population III: B220^+^IgG^−^.

**Figure 6:**
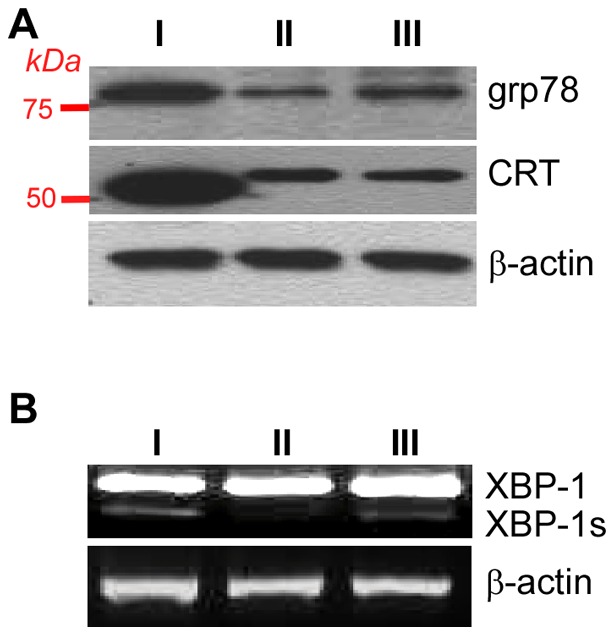
Bone marrow basophils are marked by strong unfolded protein response. *A*, Western blot for grp78 and CRT of sorted populations. *B*, RT-PCR of XBP-1 and its spliced form XBP-1s.

To determine if the generation of gp96MD is due to an alternative splicing, we next performed PCR analysis of gp96 cDNA from basophils using primers across all introns, followed by extensive sequencing analysis. We found no evidence for alternative splicing of gp96 mRNA in basophils (Fig. 5). The sequence of the full-length gp96 cDNA from basophils was identical to that from B cells (data not shown). There was no RNA editing, resulting in introduction of any new stop codons for premature termination of translation. The finding of truncation of gp96 in basophils prompted us to examine other ER HSPs in this cell type. We found that basophils expressed significantly more ER chaperones in the UPR pathway including GRP78 and calreticulin (CRT) (Fig. 6A); the latter appeared to have a faster mobility that was likely due to differences in posttranslational modification. At least one UPR sensor, the spliced form of XBP-1 (XBP-1s) [24] was induced in basophils (Fig. 6B). Thus basophils are marked not only by emergence of gp96MD post-translationally, but also by induction of UPR.

### gp96MD is generated by proteolysis

**Figure 7:**
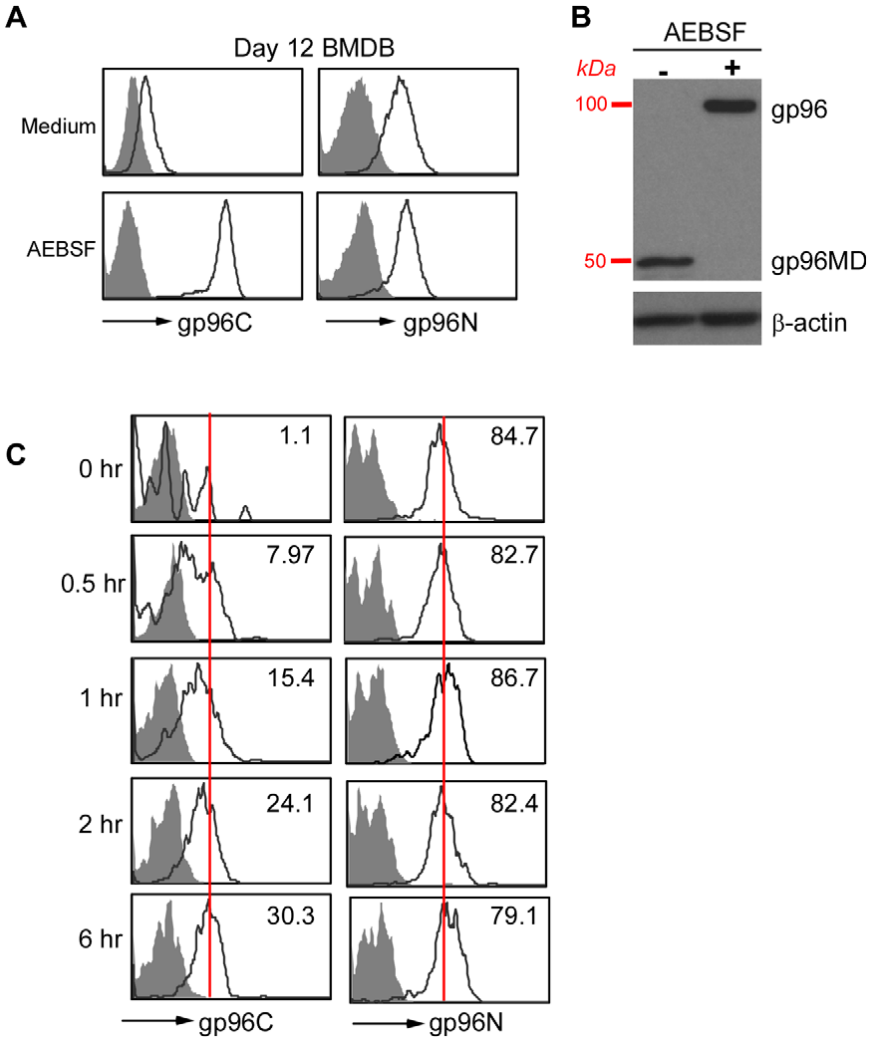
Expression of gp96MD is mediated by proteolysis. *A*, BMDBs were treated with AEBSF for 6 hours followed by intracellular stain for gp96 using either C-terminal or N-terminal specific antibody. *B*, BMDBs were treated with AEBSF for 6 hours followed by subjecting total cell lysate to electrophoresis and immunoblot for gp96. *C*, Kinetic emergence of intracellular gp96 C-terminal antibody reactivity after treatment of B220^−^IgG^+^ population with AEBSF for the indicated time. Number in the quadrant indicates mean fluorescence intensity of gp96 stain. For comparison, peak intensity of the stain at 6 hours were indicated with a vertical line.

To determine the mechanism of gp96MD generation, we derived basophils from murine bone marrow with IL-3. These FcεR^+^c-Kit^−^ bone marrow-derived basophils (BMDBs) have typical morphology with abundant and intense basophilic granules in the cytosol (data not shown). Indeed, BMDBs express no full-length gp96 evidenced by immunoreactivity to gp96N antibody but not to gp96C antibody (Fig. 7A and 7B). To examine if proteolysis is involved in the generation of gp96MD, we treated BMDBs with a variety of protease inhibitors followed by examining if the full-length gp96 expression can be restored. We found that 4-(2-aminoethyl) benzenesulfonyl fluoride hydrochloride (AEBSF), a widely used cell-permeable serine protease inhibitor [25], [26], [27], [28], is able to fully block gp96MD generation, evidenced by both intracellular stain before cell lysis (Fig. 7A) and immunoblot after cell lysis (Fig. 7B). By treating primary bone marrow cells with AEBSF kinetically, we found that gp96 expression was restored as early as 30 min after AEBSF exposure (Fig. 7C). These data indicate that full-length gp96 undergoes active proteolysis in murine basophils by serine protease(s). The fact that gp96 cleavage occurs can be observed in the absence of cell lysis excludes the possibility that the proteolysis of gp96 is an artifact that is due to exposure to proteases, as a result of loss of integrity of ER membrane.

### Human basophils do not undergo active proteolysis of gp96

**Figure 8:**
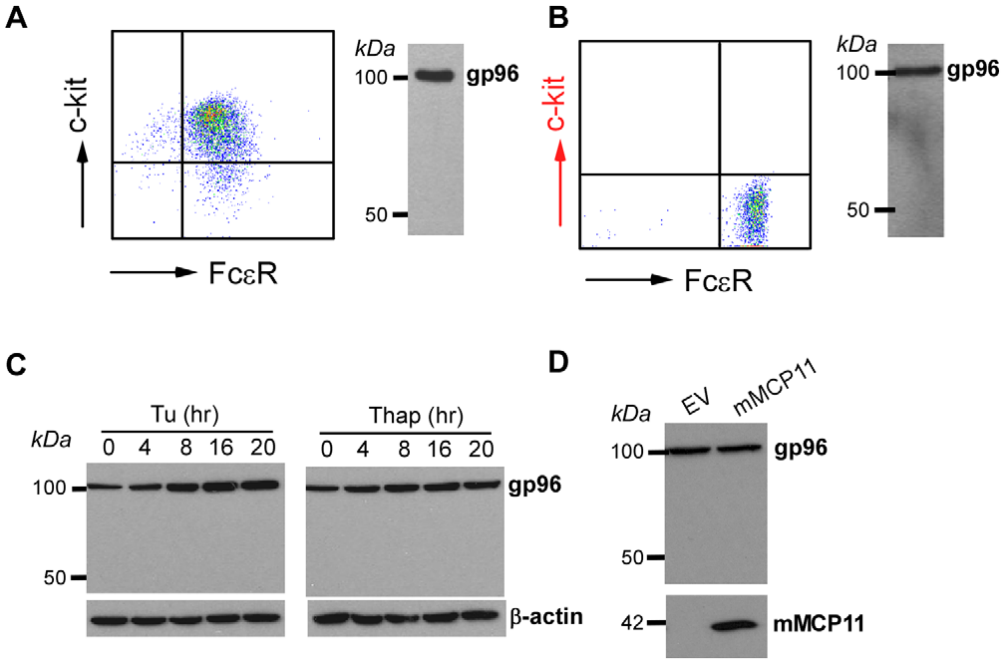
gp96MD expression is restricted to mouse basophils. *A*, Murine mast cells express intact gp96 without evidence for gp96MD. Left panel showed flow profile of bone marrow-derived mast cells. Right panel demonstrates gp96 immunoblot. *B*, Purified human basophils (left panel) only express full-length gp96 (right panel). *C*, Human KU812 basophil cell line does not cleave gp96 with and without ER stress induced by tunicamycin (Tu) or thapsigargin (Thap), demonstrated by gp96 immunoblot. *D*, Western blots for gp96 in the whole cell lysate of KU812 cells with and without ectopic expression of mMCP11.

So far we have demonstrated that gp96MD is present in murine basophils from a variety strain of mice including BALB/c, C57BL/6 and NOD. There is no evidence for gp96 cleavage in FcεR^+^c-Kit^+^ bone marrow-derived mast cells (Fig. 8A). We next looked into human basophils to determine how general gp96 fragmentation is. Surprisingly, only full-length gp96 was present in FcεR^+^ primary human basophils (Fig. 8B). Similarly, KU812, a commonly used human basophil cell line, is also deficient of gp96MD. We next turned our attention to conditions that might induce gp96 deletion. Since gp96MD expression correlated strongly with UPR, we induced UPR with standard ER stressors including tunicamycin and thapsigargin. None of the stressors is able to induce gp96 cleavage (Fig. 8C). We also considered the possibility of species-specific protease in basophils. Mouse basophils are known to express abundant serine protease mMCP11 that is absent in human basophils [29]. To determine if mMCP11 is responsible for generation of gp96MD, we overexpressed mMCP11 in human basophils. No gp96MD appearance was observed despite strong ectopic expression of mMCP11 (Fig. 8D). We conclude therefore that post-translational cleavage of gp96 is a unique process to murine basophils.

### gp96 deletion from human basophils does not grossly compromise their secretory function

**Figure 9:**
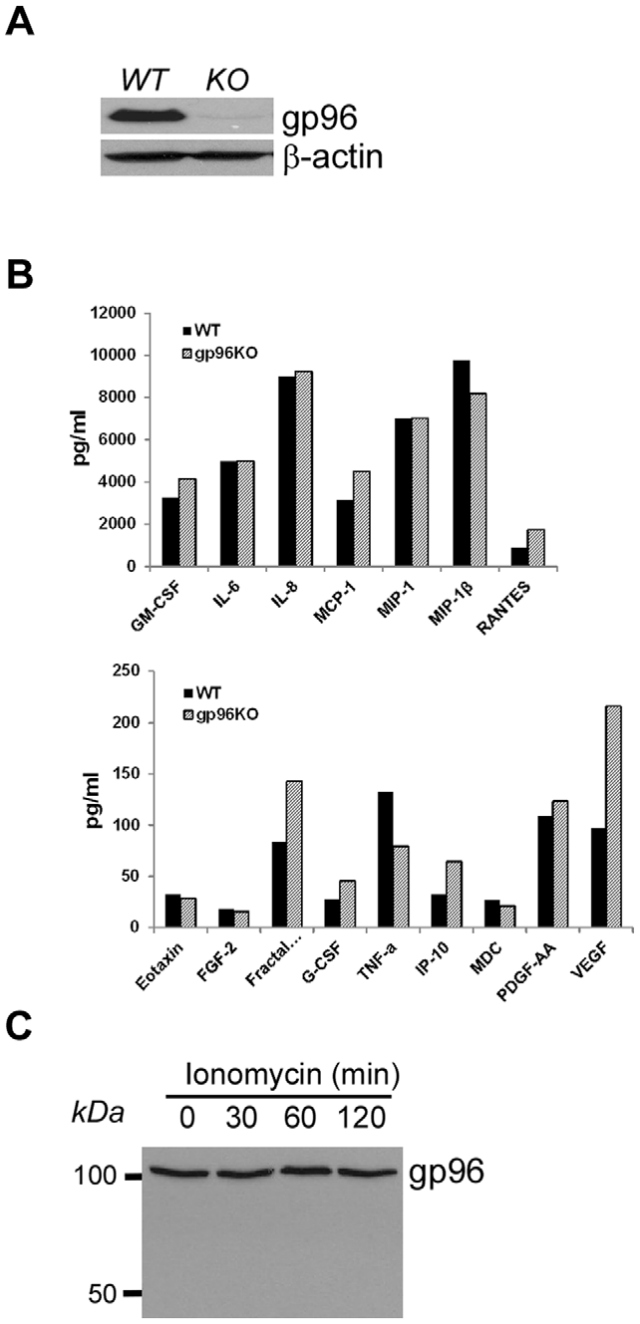
gp96 deletion does not compromise the global secretory machinery of basophils. *A*, gp96 immunoblot to demonstrate the efficient knockdown of gp96 by shRNA. *B*, Efficient cytokine and chemokine production by both *WT* and gp96 knockdown basophils in response to ionomycin. *C*, Immunoblot of gp96 after treatment with ionomycin.

Basophils have large capacity of protein synthesis and secretion via efficient protein folding and exocytosis upon activation. As a molecular chaperone in the ER, gp96 has been implicated in the quality control of general protein synthesis. To gauge the consequence of gp96 loss on the secretory function of basophils, we next knocked down gp96 from human basophil KU812 using shRNA technology (Fig. 9A). We then stimulated the gp96 knockdown cells and control cells with a calcium ionophore, ionomycin, which is an activator of human basophils [30]. A quantitative fluorescence-based cytokine muliplex assay was then performed to measure the level of a variety of secreted cytokines (Fig. 9B). We found that gp96 knockdown cells retain their ability to secrete a large array of cytokines and chemokines. In addition, we also found no evidence of gp96 cleavage after ionomycin treatment (Fig. 9C). We conclude thus that gp96 does not play significant roles in the basic secretory function and degranulation of basophils.

## Discussion

In this study, we found that gp96 is dispensable for basophil development in mice. We also made an unexpected observation that murine basophils do not express full-length gp96 due to post-translational proteolysis. Furthermore, we found that UPR was strongly induced in basophils. Our observation raises a number of intriguing questions related to the unique regulation of basophils by gp96 and UPR in murine basophils. Is gp96 shutting-down significant for basophil development and homeostasis? What is the exact nature of protease that is responsible for gp96 cleavage in basophils? How is the UPR initiated in basophils? Are gp96 loss and the emergence of gp96MD responsible for UPR in basophils? What is the functional significance of gp96 cleavage and UPR in basophils? What is the molecular and functional basis for the species-specific difference in the post-translational regulation of gp96?

We do not have answers to these questions. We have ruled out the possibility that the generation of gp96MD was due to an alternative splicing based on PCR with primers across all introns and extensive sequencing. Although stop codon read-through can occur, there is no precedent for premature translational termination at non-stop codons [31]. The bulk of the 96 kDa M.W. of gp96 is the polypeptide chain itself; there are several potential N-linked glycosylation sites, but only one site is used [32]. There are basophil-specific proteases that might play a role in gp96 cleavage. We specifically examined mMCP11 for three reasons. First, mMCP11 is a serine-protease which is expected to be inhibited by AEBSF. Second, mMCP11 is expressed in murine basophils but not in the other hematopoietic cells [29]. Third, more importantly, mMCP11 is not present in human. However, ectopic expression of mMCP11 in human basophils failed to generate gp96MD. Hellmen and colleagues have identified a protease called mMCP-8 which is uniquely expressed in mouse basophils [33], [34]. The substrate specificity of mMCP-8 has not been defined yet using the conventional substrates. The possibility of mMCP-8 to cleave gp96 to generate gp96MD remains speculative. Regardless of the mechanism of gp96MD generation, this molecule is expected to be “dead” in folding client proteins due to a loss of functionally important dimerization domain of gp96 at the C-terminus.

There is limited information on the developmental details of basophils and their relationship to other cell lineages [1], [35]. Although IL-3 is able to drive basophil differentiation [36], it is not essential for basophil development evidenced by normal number of basophils in IL-3 *KO* mice in the steady state [37]. Our finding that gp96 was inactivated in the basophils suggested that gp96 is dispensable for basophil development. Indeed, we found that the differentiation of B220^−^IgG^high^FcεRI^+^ basophils could proceed well after gp96 was efficiently deleted from the hematopoietic system. Further study of the timing of the emergence of gp96MD in the early stage of myeloid differentiation should be useful in understanding more clearly the roles of gp96 shutdown in basophil development. For example, it is unclear at this point if gp96MD appears only at a particular window during basophil development.

Given the fact that gp96 is a TLR-chaperone, shutting down gp96 might lead to attenuation of TLR signaling in basophils which likely have functional and beneficial consequence. It has been shown that basophils express both TLR2 and TLR4 mRNA, but it does not respond to LPS [21]. It is also unclear if basophil responsiveness to TLR2 ligand is truly mediated by TLR2 but not by other innate receptors, particularly since the activity could not be blocked by NF-κB inhibitor [38]. The full significance of our finding waits for the mechanism/regulation of gp96 cleavage, the knowledge of gp96MD appearance during both physiological and pathological conditions such as in situation of parasitic infection and acute allergic attack. Enforcing a protease-resistant gp96 in basophils shall be helpful to address the roles of gp96 and thus TLR in basophil development and function in the future.

What is the most surprising however is that gp96 cleavage appears to be species-specific. There is no evidence of gp96 cleavage in both primary human basophils and the established human basophil cell lines. Additionally, no emergence of gp96MD was found even after basophil activation with calcium ionophone or after classic ER stress. We have also ruled out the possibility of missing mMCP11 in human as the basis for lack of gp96 cleavage in human basophils. The striking difference in post-translational regulation of gp96 between human and murine basophils highlights the need for caution in using mouse models to infer the function of basophils in human immune response. Lee and McGarry argued elegantly that mouse perhaps has two types of basophils. The first type of “basophils” is the one that bears the following cell surface marker B220^−^IgG^+^CD19^−^IgM^−^IgD^−^CD138^−^Gr-1^−^DX5^+^FcεRI^+^CD11b^low^. These cells are abundantly present in mouse bone marrow but do not have the typical basophilic cytoplasmic granules. These basophil-like cells are the focus of almost the entire literature on mouse basophils including our current study. The second type of basophil in mouse is the morphologically defined and characteristic cells that truly resemble human basophils. But these basophils are extremely rare in mice. Thus, two questions are unanswered. First, are basophil-like cells in mice present in humans? Second, do mice have cells that are equivalent to human morphologically distinctive basophils? Our findings that mouse basophil-like cells are different from all other cells we know in its expression of gp96MD argues strongly that the phenotypically defined mouse basophils may be a distinct lineage from the morphologically defined typical basophils.

In summary, we have found that gp96 is dispensable for the development of basophil-like cells in mice and it is truncated and non-functional in these cells due to a serine-protease-dependent proteolysis. In spite of many unanswered questions, we believe that our finding is novel and significant, because, (i) it provides the first example of natural gp96 mutant in mammalian cells; (ii) it suggests unique regulation of UPR in murine basophil-like cells; (iii) it represents a unique and striking example of how two families of immune important molecules (TLRs and integrins) might be silenced coordinately and post translationally; (iv) it has uncovered a unique marker and opportunity for study of basophil-like cells in mice in the future; and (v) it uncovered a species-specific regulation of basophils which many have significant functional implications in the field of basophil biology.

## Materials and Methods

### Mice

*Hsp90b1^flox/flox^CD19^cre^* mice and control littermates have been described [12]. Tamoxifen (TAM)-inducible gp96 null mice were generated by crossing *Hsp90b1^flox^* mice with *Rosa26^ERcre^* mice (obtained from James Li, Farmington, CT) and gp96 deletion was induced reliably by 10–14 consecutive daily i.p. injection of TAM (Sigma, 100 μg/20 g body weight in peanut oil) [15], [17]. Mice were bred and maintained according to the established guidelines and an approved protocol by Medical University of South Carolina Institutional Amimal Care and Use Committee. All other mice were obtained from Jackson Laboratories (Bar Harbor, ME).

### Antibodies and other reagents

Antibodies (Ab)s against GRP78 and gp96 were purchased from Stressgen (Victoria, BC, Canada). Calreticulin Ab was purchased from BD Bioscience (Mountain view, CA). All Abs used for flow cytometry were obtained from BD Bioscience (Mountain view, CA) and eBioscience (San Diego, CA).

### RT-PCR

mRNA isolation, reverse transcription and sequence-specific amplification of cDNAs for Igα, gp96, IL-4 and XBP-1 were performed with the standard protocol, using the following primers: 5′-ATGAGGGCCTGAACCTTGATGACT-3′ (forward) and 5′-CAAGGGCTGCTTTGGGAAGGATTT-3′ (reverse) for Igα, 5′-TCACTGACGGCACAGAGCTATTGA-3′ (forward) and 5′-AATATGCGAAGCACCTTGGAAGCC-3′ (reverse) for IL-4; and 5′-ACACGCTTGGGAATGGACAC-3′ (forward) and 5′-CCATGGGAAGATGTTCTGGG-3′ (reverse) for XBP-1 [39].

### Cells, retroviral expression vectors

gp96 mutant pre-B cell lines were obtained from Brian Seed (Harvard University, Boston, MA). Murine mast cells were derived *in vitro* from bone marrow precursors using the standard protocol [40]. Full-length gp96 cDNA and gp96 deletion mutants were cloned into the MigR retrovector by a PCR-based strategy. Virus propagation and transduction of pre-B cells were based on the established protocols [11]. mMCP11 retroviral expression vector DNA was a kind gift from Toshiyuki Kojima (Tokyo Medical and Dental University, Japan).

### Flow cytometry, FACS sorting and multiplex cytokine assay

Cell staining and flow cytometry were done as described [12]. The bone marrow basophils were purified by FACS sorting on a BD Biosciences (San Jose, CA) FACSVantage SE with DiVa option. Multiplex cytokine assay was performed by Eve Tech (Calgary, Alberta, Canada)

### Protein extraction and Western blot

Protein extraction and immunoblot were performed as described previously [12]. Briefly, cells were washed three times with ice-cold PBS and lysed in radioimmunoprecipitation assay (RIPA) lysis buffer (0.01 M sodium phosphate, pH 7.2, 150 mM NaCl, 2 mM EDTA, 1% NP-40, 1% sodium deoxycholate, 0.1% SDS, 2 mM AEBSF, 130 mM bestatin, 14 mM E-64, 0.3 mM aprotinin, and 1 mM leupeptin). Total cell lysates was resolved on denaturing and reducing 10% SDS-PAGE, and the proteins were transferred from the gel onto Immobilon-P membranes. The membrane was blocked with 5% nonfat milk in PBS and then incubated with different Abs, followed by incubation with HRP-conjugated secondary Ab. Protein bands were visualized by using enhanced chemiluminescent substrate (Pierce, Rockford, IL).
